# Evaluating laboratory cyberbiosecurity capacity in Morocco: development and pilot implementation of the cyber self-laboratory assessment tool (Cyber-SLAT)

**DOI:** 10.3389/fbioe.2026.1947925

**Published:** 2026-10-21

**Authors:** Alexander G. Linder, Vanessa Grégoire, Larbi Baassi, Sanae Lemrabet, Erin M. Sorrell

**Affiliations:** 1 Department of Environmental Health and Engineering, Bloomberg School of Public Health, Center for Health Security, Johns Hopkins University, Baltimore, MD, United States; 2 Biorisk Management Association of Morocco (BMAM), Rabat, Morocco

**Keywords:** biorisk management, cyberbiosecurity, Cyber-SLAT, information security, laboratory assessment

## Abstract

**Introduction:**

Cyberbiosecurity, the discipline addressing risks at the convergence of biological research and digital infrastructure, has advanced conceptually but remains largely untranslated into laboratory practice, and empirical assessments of laboratory‐level cyberbiosecurity capacity in low‐ or middle‐income countries (LMICs) remain rare.

**Methods:**

The objective of this study was to develop and pilot the Cyberbiosecurity Self‐Laboratory Assessment Tool (Cyber‐SLAT), which integrates a new cyberbiosecurity domain of 57 questions into an existing, validated biorisk management self‐assessment instrument, yielding a bilingual (English/French) tool of 258 questions across six domains that span risk evaluation, planning and management, infrastructure and systems, and incident response. The Cyber‐SLAT was piloted across seven Moroccan laboratories representing five institutional types, using an implementation‐driven design that combined virtual training, structured self-assessment, quantitative gap analysis, a review of corrective actions, and a two-day in-person capacity‐building dialogue that included the national cybersecurity authority.

**Results:**

While traditional biorisk management functions were generally operational, the cyberbiosecurity domain contained the highest concentration of gaps of any domain assessed (53 of 129 identified problem questions). Two universal gaps, which no laboratory could affirm, and 12 critical gaps were identified related to cyberbisoecurity, with 12 of these 14 severe gaps (86%) addressing operational rather than technical capacities. These findings suggest that the principal barrier for laboratories entering the cyberbiosecurity domain is operational rather than technical, and that operational foundations must be established before technical safeguards can be effectively implemented or sustained. The assessment process was accompanied by a cross-functional dialogue between laboratorians and information technology personnel and established direct linkages between the laboratory community and national cybersecurity governance.

**Discussion:**

This pilot provides, to our knowledge, the first application of the full Cyber‐SLAT in North Africa, and adds to a small but growing evidence base on laboratory cyberbiosecurity capacity in resource‐constrained settings.

## 1 Introduction

Cyberbiosecurity is a rapidly evolving field that addresses the unique vulnerabilities emerging at the interface of biological research and information technology (Murch et al., 2018). As laboratory operations have grown dependent on digital infrastructure, including laboratory information management systems (LIMS); networked scientific instruments; cloud-based data storage platforms; Internet of Things (IoT) devices; and automated sequencing and synthesis equipment, new categories of risk have emerged that neither cybersecurity nor biosecurity frameworks adequately address in isolation. The physical-digital interface, where physical laboratory equipment and processes interact with digital tools, systems, and software, represents a critical attack surface unique to laboratory settings (Richardson et al., 2019). Information systems, including LIMS, electronic health records, and genomic databases, store sensitive data on pathogen types, locations, and transport that could be exploited if inadequately secured (Mueller, 2021). These risks are compounded by advancements in dual-use technologies such as artificial intelligence, next-generation sequencing, synthetic biology, and cloud-based laboratory automation, that expand both the capabilities and the attack surface of modern laboratories. The increasing volume, sensitivity, and interconnectedness of biological data generated by modern research enterprises create persistent data stewardship challenges.

Cyberbiosecurity has accordingly gained institutional traction and has been named an “urgent priority” by organizations like the European Union Agency for Cybersecurity and United Nations Institute for Disarmament Research (European Union Agency for Cybersecurity, 2022; Mazeaud and Kastelic, 2025). In 2025, UNIDIR published a policy brief arguing that cyberbiosecurity “deserves more attention in multilateral forums” (Mazeaud and Kastelic, 2025). Two widely recognized information security frameworks have emerged as reference standards for cyberbiosecurity implementation (Cova et al., 2026): the International Organization of Standardization (ISO) 27,001/27,002 (International Organization for Standardization, 2022), which provides requirements for establishing, implementing, and maintaining an information security management system (ISMS), and the United States National Institute of Standards and Technology (NIST) Cybersecurity Framework 2.0 (NIST, 2024), which organizes cybersecurity risk management around five core functions—Identify, Protect, Detect, Respond, and Recover. Both frameworks offer structured approaches to managing digital risks in laboratory environments, though neither was designed specifically for the intersection of cybersecurity and biosecurity.

Despite growing recognition as a distinct discipline by policymakers and technical experts, cyberbiosecurity suffers from a significant gap between its conceptual development and its translation to operational settings. The field remains dominated by theoretical frameworks, taxonomies of hypothetical threats, and proof-of-concept demonstrations (Elgabry and Johnson, 2024). Much of this literature focuses on technically sophisticated scenarios such as DNA-encoded malware, adversarial attacks on protein prediction models, and nation-state espionage on vaccine research (Liu et al., 2024). While real concerns, these threats have limited applicability to the day-to-day realities of laboratories conducting critical diagnostics and advanced biological research. The laboratory, which is the actual nexus of both biological research and cyberbiosecurity risk, has received remarkably little empirical attention. No formal assessment has measured how laboratories perform across cyberbiosecurity domains, what specific challenges they face, or how these vary by institutional type, resource level, or geographic context. Further, the convergence of biological and digital systems presents a fundamental governance challenge. Existing laboratory oversight structures were designed to manage physical and biological risks, not digital ones. Biosafety officers were trained to contain pathogens and provide a safe working environment for researchers; information technology (IT) departments to protect enterprise networks; researchers to prioritize data openness and reproducibility. Cyberbiosecurity cuts across all three domains but sits neatly within none of them and must be considered a new category of risk that existing institutional roles must now absorb without clear precedent for how responsibility should be allocated.

The above claims are supported by existing literature: empirical evidence on cyberbiosecurity assessments in laboratory settings remains particularly thin. A recent PRISMA-conducted scoping review of cyberbiosecurity implementation in laboratory settings found that cyberbiosecurity literature remain dominated by reviews and policy papers rather than primary studies: of 30 articles analyzed, only two were primary implementation studies; 63% focused on high income countries; and guidance for clinical diagnostic laboratories was underrepresented (Cova et al., 2026). The review concluded that no standardized approaches currently exist for assessing or implementing cyberbiosecruity capacities in laboratory facilities. Laboratories seeking to assess cyberbiosecurity-relevant capacities today must choose among instruments that are either generic or specific to biorisk management without cyberbiosecurity content or vice versa. The NIST Cybersecurity Framework 2.0 (NIST, 2024) and ISO/IEC 27001:2002 provide structured approaches to information security management but assume dedicated staffing and, in the case of ISO standards, substantial costs for continued certification. Laboratory biorisk managements tools such as the Biosafety and Biosecurity Laboratory Assessment Tool (BSS LAT; Orelle et al., 2022) is a freely available, multilingual, spreadsheet-based self-assessment with automatic scoring that has been tested in several countries, and Sandia National Laboratories’ BioRAM software (Sandia National Laboratories, 2021) supports structured laboratory biorisk assessment under restricted access. Neither, however, contains cyberbiosecurity content. A feature level comparison of these frameworks and tools is provided in Supplementary Table S1.

Despite low levels of evidence in the published literature, efforts are growing to integrate cyberbiosecurity into international biorisk assessments. The World Health Organization, 2024 Laboratory Biosecurity Guidance represents a paradigm shift by explicitly integrating cybersecurity into biorisk management frameworks and recommending that Institutional Biosafety Committees (IBC) expand their role to include oversight of information security risks (World Health Organization, 2024). This guidance, however, is somewhat aspirational rather than prescriptive: no implementation models exist to emulate, no tools have been validated for assessing cyberbiosecurity capacity in resource-constrained settings, and no country has demonstrated how to translate international guidance into operational laboratory practice.

The translation gap between the theoretical framing of cyberbiosecurity combined with the lack concrete tools for assessment and guidance takes on particular significance in low- and middle-income country (LMIC) settings, where biological research capacity is expanding rapidly and laboratories are adopting the same networked digital infrastructure that generates cyberbiosecurity risk. The governance architectures, resource environments, and institutional configurations in these settings differ substantially from the contexts in which cyberbiosecurity frameworks were developed. The field’s predominantly Western origins, rooted in United States national security concerns and European regulatory frameworks, mean that its core assumptions about institutional capacity, threat models, and regulatory infrastructure do not translate directly (Fouad, 2024). This is not a question of LMIC laboratories lagging behind a universal standard; it is a question of a field that has not yet produced the practical tools, implementation models, or empirical evidence needed to operate across diverse institutional contexts. Established frameworks such as ISO 27001/02 and the NIST Cybersecurity Framework provide structured approaches to information security management, but their application to laboratory cyberbiosecurity in low- and medium-resource settings remains underassessed, and no guidance exists on how laboratories should map their specific cyberbiosecurity vulnerabilities to the controls and functions articulated in these standards. While standardized assessment tools exist for traditional laboratory biorisk management in LMICs (Ong et al., 2025; Orelle et al., 2022) no equivalent instrument within such tools addresses cyberbiosecurity, and empirical data on laboratory-level cyberbiosecurity capacity in LMIC settings remain scarce, though efforts to assess cyberbiosecurity in public health laboratories as a component of biorisk management from the Democratic Republic of the Congo (DRC) demonstrate a growing acknowledgment of the importance of cyberbiosecurity concerns in LMICs (Cova et al., 2026; Kambama-Tshiknogo et al., 2026).

This effort aims to build cyberbiosecurity evidence base in LMICs and involved an empirical assessment of cyberbiosecurity capacity in the Kingdom of Morocco (Morocco), a middle-income country with a growing laboratory research sector, established biosafety governance structures, and increasing integration of digital technologies into laboratory operations. Specifically, this work aimed to:

1. Develop a cyberbiosecurity self-laboratory assessment tool (Cyber-SLAT) integrated into an existing biorisk management assessment framework, tailored for laboratories in low- and medium-resource settings;
2. Pilot the Cyber-SLAT across a diverse sample of Moroccan laboratories to systematically identify cyberbiosecurity vulnerabilities, gaps, and areas for improvement;
3. Characterize patterns in cyberbiosecurity readiness across laboratory types, assessment domains, and severity levels, with particular attention to distinguishing organizational from technical gaps; and
4. Document a replicable capacity-building methodology that combines structured assessment with targeted training and action planning for resource-constrained settings.

To our knowledge, this represents the first pilot of the full 57-question Cyber-SLAT cyberbiosecurity domain administered as a laboratory self-assessment with integrated severity grading and action planning and the first such effort in North Africa.

## 2 Materials and methods

This study employed a implementation science approach combining tool development, structured self-assessment, quantitative gap analysis, review of corrective actions, and a capacity-building intervention. The project was conducted between March and December 2025.

### 2.1 Tool development

The Cyber Self-Laboratory Assessment Tool (Cyber-SLAT) was developed by modifying an existing Self-Laboratory Assessment Tool (SLAT) (GU SLAT). The original GU SLAT was designed to assess biorisk management functions in public health, environmental, and veterinary laboratories and was specifically tailored to address common challenges observed in low- and medium-resource settings where international biosafety guidance documents often lack implementation specificity (Ong et al., 2025) The GU SLAT includes 201 questions across five assessment domains organized as tabs within a spreadsheet-based tool: (1) Infrastructure and Equipment (43 questions), (2) Administration and Training (29 questions), (3) Biorisk Management (35 questions), (4) Specimen Management (46 questions), and (5) Waste Management (48 questions). Each tab is further divided into subsections corresponding to specific functional areas.

Under this effort, the GU SLAT was revised to incorporate cyberbiosecurity considerations through the addition of a sixth assessment domain, bringing the newly amended Cyber-SLAT to 258 questions across six tabs (Table 1). The cyberbiosecurity domain was developed collaboratively between the Johns Hopkins Center for Health Security and Georgetown Center for Global Health Science and Security, drawing on the gaps identified in a scoping review of cyberbiosecurity implementation by Cova and colleagues (Cova et al., 2026) as well as an exploratory review of publicly available peer-reviewed and grey literature on cyberbiosecurity and related concepts, including cybersecurity, data privacy, and laboratory information security. Key themes and standards were identified and extrapolated into assessment questions that were validated by subject matter experts from the US and EU. The resulting cyberbiosecurity section consists of 57 questions organized across four sections and 12 subsections. These include: Section 6.1 Risk Evaluation (12 questions) covers high-risk activities and partnerships and research collaborations; Section 6.2 Planning and Management (17 questions) addresses roles and responsibilities, key documentation and policies, and acceptable use; Section 6.3 Infrastructure and Systems (24 questions) encompasses network and systems administration, access controls, IT systems, cloud and data storage, and data stewardship; and Section 6.4 Incident Response (4 questions) assesses preparedness for cyberbiosecurity events (Linder et al., 2026).

**TABLE 1:** Cyber-SLAT assessment domains and question distribution.

Assessment tab	Tab subsections	Questions
1. Infrastructure & equipment	Infrastructure management; general condition; laboratory ICT; utilities; equipment inventory, use, maintenance, monitoring	43
2. Administration & training	Administration and management; quality management; training	29
3. Biorisk management	BRM administration; biorisk assessment; biorisk mitigation; BRM implementation and operation; BRM performance review	35
4. Specimen management	Specimen administration; specimen inventory; specimen requests; specimen collection; specimen handling and receipt; specimen referral and transport	46
5. Waste management	Waste management procedures; waste management biosafety; waste management policies; waste segregation and storage; waste management disinfection; waste disposal and transport	48
6. Cyberbiosecurity	Risk evaluation; planning and management; infrastructure and systems; incident response	57
Total	​	258

The four sections were designed to capture a comprehensive picture of laboratory cyberbiosecurity capacity, progressing from risk awareness (Section 6.1); organizational governance and policy (Section 6.2); technical infrastructure and controls (Section 6.3); and preparedness for and response to cyberbiosecurity incidents (Section 6.4). The tool includes a user guide with definitions of key cyberbiosecurity terms, guidance on which personnel should contribute to each section, and a glossary of relevant acronyms. Each question can be answered using a standardized response format: “Yes,” “No,” or “Not Applicable” (N/A). For each question, respondents are also provided fields to document proposed corrective actions and comments providing additional context. A final tab within the tool aggregates responses and enables identification of priority corrective actions through a generated action plan available in the tool, drawing from responses for all previous tabs.

The complete Cyber-SLAT is available as an English/French spreadsheet-based instrument that can be completed electronically or printed for manual completion (Linder et al., 2026). The spreadsheet-based format was a deliberate design choice for low-resource settings: the tool requires no installation of specialized software apart from basic spreadsheet tools, requires no internet connectivity, can be printed to be completed by hand, and can be adapted or translated without programming expertise. Although the format could in principle be adapted into a dedicated software system, it was retained for this pilot to prioritize ease and use and accessibility for laboratories with limited information technology infrastructure. The French version of the tool was reviewed for linguistic and cultural appropriateness by Moroccan collaborators. The complete Cyber-SLAT is downloadable without restriction, and the developement team welcomes feedback and recommendations for future revisions and iterations (Linder et al., 2026; Ong et al., 2025).

### 2.2 Study setting and laboratory selection

Seven Moroccan laboratories were selected for participation through a collaborative process between Johns Hopkins University Center for Health Security (JHU-CHS) and the Biorisk Management Association of Morocco (BMAM). BMAM is the premier biosafety professional association dedicated to promoting biorisk management practices across Moroccan research and diagnostic facilities, allowing for the building of trusted relationships across the project.

Laboratories were selected to represent the diversity of institutions engaged in advanced biological research in Morocco, including facilities conducting synthetic biology, critical diagnostic testing, and advanced genomic research. Selection followed explicit criteria and constraints, and the participation of seven facilities across five institutional types reflects available project funding and the engagement-intensive nature of the capacity-building model. Selection prioritized high-containment laboratories and/or those with United States research partnerships (Table 2). The sample size is therefore purposive and not statistically representative of the Moroccan landscape.

**TABLE 2:** Participating laboratory characteristics.

Lab ID	Institutional type
1	Private clinical diagnostic laboratory
2	University research laboratory
3	National reference laboratory
4	Regional analytical and research laboratory
5	Regional hospital laboratory
6	Veterinary research laboratory
7	Private pharmaceutical laboratory

Despite these constraints, this sample allowed the project to assess cyberbiosecurity challenges across different institutional contexts, resource levels, and governance structures. To protect participating facilities, institutional characteristics are reported without laboratory identifiers, and results are presented in aggregate or under arbitrary anonymized laboratory codes that were rerandomized for publication. Individual laboratory data is not available.

### 2.3 Assessment process

The assessment was implemented through a structured three-phase process: virtual training and self-assessment period, data analysis, and in-person capacity building.

#### 2.3.1 Virtual training and self-assessment period

In June 2025, JHU-CHS and BMAM convened a virtual training to introduce participating laboratories to the Cyber-SLAT tool and provide foundational instruction on cyberbiosecurity concepts. Event participants included laboratory directors, biosafety officers, IT systems administrators, laboratory technicians, and researchers from the seven participating laboratories. The training consisted of plenary sessions covering: (a) an introduction and overview of the Cyber-SLAT, including the assessment domains and completion instructions; (b) a cybersecurity and cyberbiosecurity overview, including the newly introduced cyberbiosecurity content areas; and (c) a group discussion on best practices for completing the assessment and prioritizing corrective actions.

Following the virtual training, participating laboratories completed Cyber-SLAT self-assessments over a period of approximately 6 weeks with in-person support provided by BMAM as needed. The assessment process required laboratories to assemble multidisciplinary teams to complete the tool. For the traditional biorisk management sections (Tab 1-5), laboratory directors and biosafety officers typically served as primary respondents, drawing on their knowledge of laboratory operations, policies, and procedures. For the cyberbiosecurity section (Tab 6), laboratories were encouraged to engage information technology staff, including network administrators, database administrators, and IT support specialists (when available), who could provide accurate information about technical controls, system configurations, and data management practices. Responses were developed through internal consensus within each laboratory team, with disagreements adjudicated internally. Documented evidence was not required to support “Yes” responses, consistent with the instrument’s design as a self-assessment tool; BMAM personnel, however had access to each participating facility’s assessment team and were available to support interpretation of assessment items throughout the assessment period. All seven laboratories successfully submitted completed assessments by the deadline, achieving a 100% completion rate.

### 2.4 Data analysis

#### 2.4.1 Quantitative analysis

Analysis of the completed Cyber-SLAT assessments focused primarily on the cyberbiosecurity tab (Tab 6), as this represented the novel component of the Cyber-SLAT. For the quantitative analysis, response distributions of “Yes,” “No,” and “N/A” were tabulated across all seven laboratories and organized by section and subsection. For this assessment, a laboratory was considered to have a capacity gap on a question when it could not affirm the capacity, that is when it responded “No” or “N/A.” This precautionary treatment was adopted for capacity-planning purposes; we acknowledge that “N/A” responses can reflect several distinct situations, including questions that are genuinely not applicable (such as functions managed by a parent institution above the laboratory’s visibility), as well as a cases in which respondents could not determine whether a capacity exists. In either case, the laboratory could not confirm that the capacity is in place, and the item represents a potential area for improvement that the laboratory may wish to consider in their action planning. This approach errs towards identifying improvement areas rather than estimating deficiencies precisely; the resulting counts should be read an upper bound or “worse case” estimate of cyberbiosecurity gaps. During the in-person dialogue, participating laboratories reviewed the assessment findings and endorsed this interpretation, using it for future action planning described below.

To prioritize findings for action planning, a severity classification was applied based on the number of laboratories that could not affirm each question. Questions that no laboratory could affirm (7/7) were classified as universal gaps. Questions that six of the seven laboratories could not affirm (6/7) were classified as critical gaps. Additional severity levels (high: 5/7; moderate: 4/7; and low 3/7) characterized the distribution of gaps across the assessment, and a question was considered a “a problem question” when three or more of seven laboratories could not affirm it. Comparative analysis across all six assessment tabs was conducted to contextualize the cyberbiosecurity findings relative to traditional biorisk management domains. We would like to acknowledge that in the Cyber-SLAT assessment a “Yes” response is not inherently favorable for every question (for instance, the ability for an outside individual to gain access to laboratory systems would be a detriment). We acknowledge this gap and are developing the inclusion of a hazard metric for each question in the second version of the Cyber-SLAT tool for a more nuanced assessment. As this effort involved the pilot version of the tool, this hazard assessment is not present in our current analysis.

Each assessment question was classified as either “Operational” or “Technical for this assessment. Operational questions address roles and responsibilities, policies, training, documentation, plans, agreements, and oversight functions. Technical questions address systems, configurations, implemented controls, and technical practices and capabilities. It should be noted that with a sample size of seven laboratories, all analyses were descriptive and were grounds for further discussion of potential capacity-building efforts at the in-person dialogue. No inferential comparison between laboratories was attempted.

#### 2.4.2 Corrective action analysis

A joint assessment examined the cyberbiosecurity-related corrective actions proposed by laboratories in the action planning section of the Cyber-SLAT. The analysis categorized corrective actions by the relevant Tab 6 subsection, assessed their assigned priority levels and estimated implementation timelines, and identified the responsible parties designated for implementation. Corrective action patterns were analyzed across laboratories to identify shared priorities and cross-referenced with the quantitative results to assess whether laboratories were proposing actions that addressed the most critical gaps identified in the assessment. No pre-or post-intervention evaluations were conducted, as the effort was designed to identify cyberbiosecurity gaps for capacity-building efforts, rather than a training on the Cyber-SLAT tool itself.

### 2.5 In-person dialogue and action planning

Following the analysis of laboratory responses, JHU-CHS and BMAM convened an in-person dialogue in Rabat, Morocco. The dialogue brought together laboratory and IT focal points from all seven participating facilities as well as two cybersecurity representatives from Morocco’s General Directorate of Information Systems Security (DGSSI), the national authority responsible for cybersecurity governance. The two-day agenda was structured around plenaries, case studies, and facilitated group discussion and work covering: assessment findings review, including laboratory-specific SWOT (Strengths, Weaknesses, Opportunities, Threats) presentations; formal information security management standards (ISO/IEC 27001 and NIST Cybersecurity Framework); risk assessment exercises using a likelihood-impact methodology (ISO/IEC 27005); action planning with linkages to international standards; data stewardship case studies on vulnerability mapping; strategic plan development; integration of cyberbiosecurity into biorisk management training; and leadership buy-in strategies.

Briefly, for the SWOT analysis, participants worked collaboratively to understand internal strengths and weaknesses and the external threats and opportunities across their facilities that shape current and future cyberbiosecurity operations. Corrective actions were collected across each of the seven laboratories’ Cyber-SLAT assessments and compared to identify the shared priorities and most critical gaps. This analysis was then applied to develop short and long-term goals that were specific and measurable targets. These SMART goals were applied to a strategic framework and action plan; each goal included activities, outputs, and indicators that were tangible, specific, and measurable. These action plans are currently being implemented across all seven laboratories in an 18-month period of performance.

## 3 Results

### 3.1 Overall assessment profile

All seven participating laboratories completed the full Cyber-SLAT assessment, yielding complete data across all six assessment tabs (258 questions total). Figure 1 presents the distribution of “Yes,” “No,” and “N/A” responses by laboratory across all tabs with laboratories identified by arbitrary, anonymized codes. Three laboratories demonstrated the strongest overall profiles, each recording over 200 “Yes” responses, suggesting relatively strong alignment across both traditional biorisk management and cyberbiosecurity domains, particularly noteworthy given that cyberbisoecurity was a novel concept for most participants. Laboratory LD was a clear outlier, recording the fewest “Yes” responses (63) and the highest proportions of “No” (155), while Laboratory LC demonstrated the most “N/A” responses (51). These divergent profiles most likely reflect institutional structure, where data security, IT governance, and infrastructure management are often overseen by higher-level authorities rather than at the laboratory level, resulting in “N/A” responses for questions where the laboratory lacks direct control or visibility.

**FIGURE 1:**
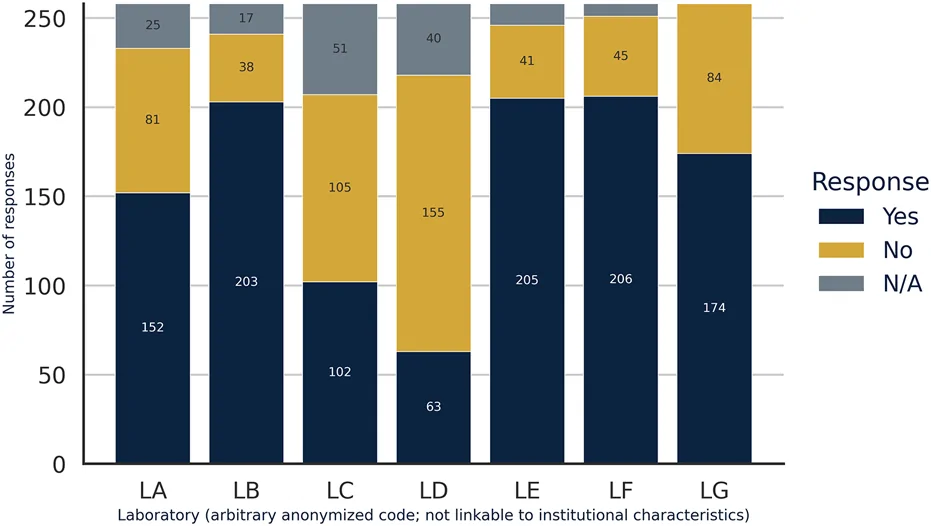
Distribution of Cyber-SLAT responses across seven Moroccan laboratories: with three response categories: Yes (navy), No (gold), and Not Applicable (grey). Laboratories appear under arbitrary, anonymized codes (LA-LG) that were re-randomized for publication and cannot be linked to institutional characteristics. Three laboratories (LB, LE, LF) recorded over 200 Yes responses each. Laboratory LD was a clear outliner (fewest Yes: 63; most No: 155), and Laboratory LC recorded the most N/A responses (51); such divergent profiles are consistent with institutional structures in which data security and IT governance managed above the laboratory level.

Comparison of response distributions across the six assessment tabs revealed that Tab 6 (Cyberbiosecurity) contained the highest concentration of gaps (Figure 2). Of 129 total “problem question,” across the entire Cyber-SLAT, that is, questions that three or more of the seven laboratories could not affirm (answered “No” or “N/A”), Tab 6 accounted for the largest share (53 questions), including both universal gaps identified in the cyberbisoecurity domain and the majority of critical gaps. The only other universal gaps responses occurred in Tab 5 (Waste Management), and were limited to two questions in the waste management disinfection subsection (5.5).

**FIGURE 2:**
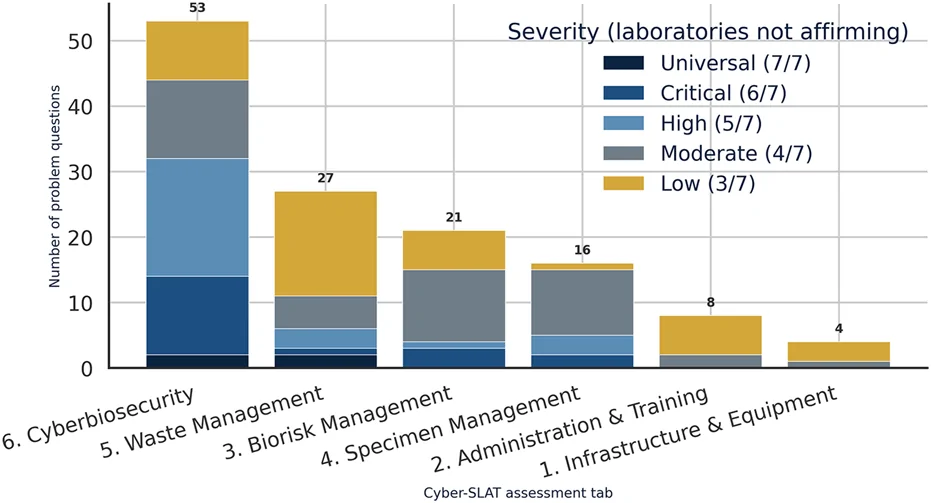
Distribution of problem questions across the six Cyber-SLAT assessment tabs, stratified by severity: of the 129 problem questions across the Cyber-SLAT, Tab 6 (Cyberbiosecurity) accounted for the largest share (53 questions), including both universal gaps identified in the cyberbiosecurity domain and the majority of critical gaps. The only other universal gaps occurred in Tab 5 (Waste Management), limited to two questions in the waste disinfection subsection (5.5).

### 3.2 Cyberbiosecurity capacity by domain

Within the cyberbiosecurity tab, response patterns varied substantially across the four sections (Figure 3). Our assessment does however explore the cyberbiosecurity sections below.

**FIGURE 3:**
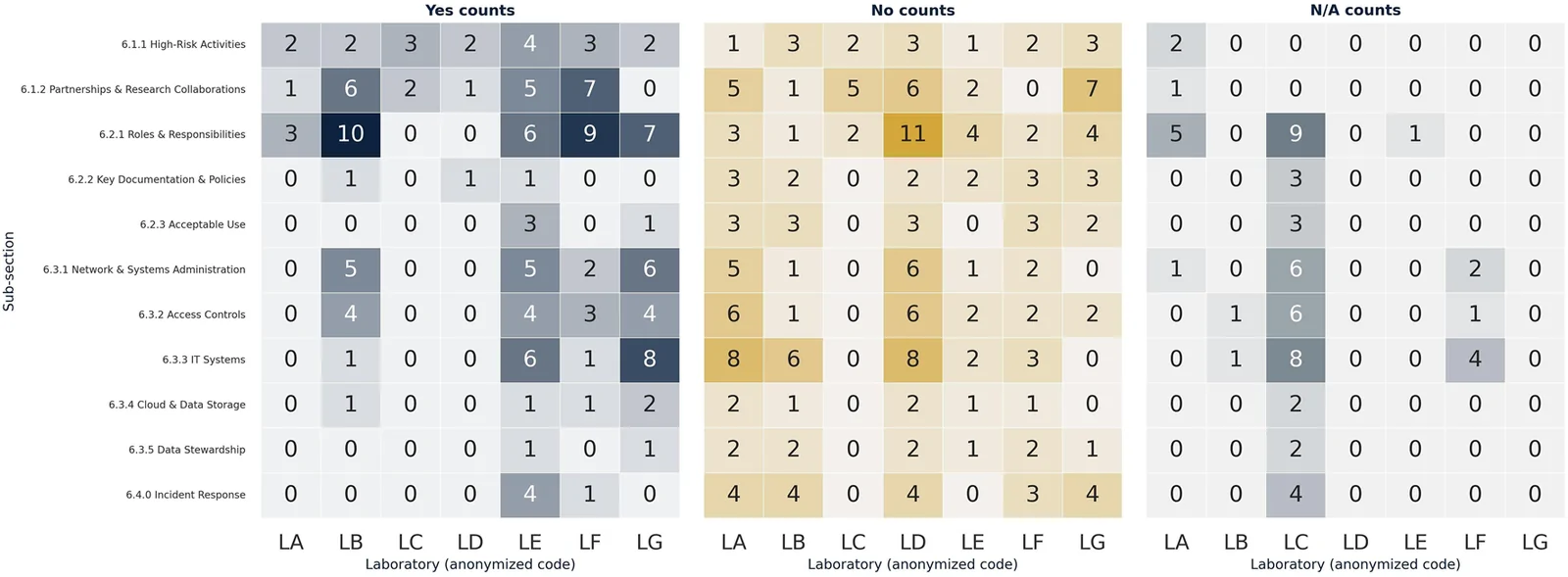
Heatmap of cyberbiosecurity (Tab 6) question responses by sub-section and laboratory shown as side-by-side heatmap: Presenting the three responses together demonstrate a cluster of “No” responses in Roles and Responsibilities (6.2.1) IT systems (6.3.3), and Partnerships and Research Collaborations (6.1.2) across most laboratories, while “N/A” responses concentrate within one laboratory (LC) across IT systems (6.3.3) Networks and Systems Administration (6.3.1) and Access controls (6.3.2).

#### 3.2.1 Risk evaluation (section 6.1, 12 questions)

Risk Evaluation was the relative strength across the sample, with most laboratories providing positive responses. Laboratories generally reported conducting diagnostics for high-consequence pathogens, performing sequencing, utilizing pathogen inventory systems, and maintaining formal partnerships with data-sharing agreements. That Risk Evaluation emerged as the strongest domain is consistent with the fact that these questions largely address activities and relationships that laboratories already manage as part of routine operations, such as diagnostics, sequencing, specimen exchange, and research collaborations, rather than requiring a cyberbiosecurity-specific lens. In effect, laboratories performed well on questions where existing biosecurity knowledge could be applied, even as they were newly encountering the concept of cyberbiosecurity as a distinct discipline. However, one universal gap was identified in this section: no laboratory could affirm having identified key hazards at the physical-digital interface of their facility (6 laboratories responded “No,” 1 laboratory responded “N/A”) This finding indicates that while laboratories are aware of their biological risks and research collaborations, they have not extended hazard identification to the points where physical laboratory processes interact with digital systems.

#### 3.2.2 Planning and management (section 6.2, 17 questions)

Planning and Management contained significant gaps concentrated in operational functions. Critical gaps (6/7 laboratories could not confirm) were identified for: designated cyberbiosecurity personnel (Q6.2.1.2), regular staff training on cyber, data, and information security (Q6.2.1.11), cyberbiosecurity threat mitigation SOPs or policies (Q6.2.2.1), and training on risks from IoT devices, generative AI, and online platforms (Q6.2.3.2), as well as training on risks of connecting personal devices to enterprise networks (Q6.2.3.3). These findings indicate that the organizational structures, personnel assignments, documentation, and workforce development necessary to embed cyberbiosecurity into routine laboratory operations are broadly absent.

#### 3.2.3 Infrastructure and systems (section 6.3, 24 questions)

Infrastructure and Systems displayed the most widespread gaps across subsections. Critical gaps (6/7 laboratories could not affirm) were identified for: restricted web browser and application access (Q6.3.2.5), utilization of recognized security standards for data storage and transfer, end-to-end data flow mapping for laboratory and pathogen data (Q6.3.5.1), and visibility into how LIMS data may be accessible via other health information systems (Q6.3.5.2). The concentration of gaps in access controls, data storage standards, and data stewardship subsections indicates that even where laboratories have basic IT infrastructure in place, critical safeguards for protecting sensitive biological data are inconsistently applied.

#### 3.2.4 Incident response (section 6.4, 4 questions)

Incident Response was the weakest domain across all laboratories, though this finding should be interpreted in the context of the section’s small question count (4 questions). No laboratory could affirm having a cyberbiosecurity incident response plan (Q6.2.2.2, Classified under Planning and Management; 7/7 could not affirm), and the section’s three remaining gaps were critical: absence of law enforcement notification processes in incident response plans (Q6.4.0.2), no evaluation policies or after-action reviews for cyberbiosecurity incidents (Q6.4.0.3), and no mechanisms for disseminating lessons learned from incidents (Q6.4.0.4). The fourth question in the section (Q6.4.0.1) discussing familiarity with law enforcement, was not affirmed by five of seven laboratories. The near uniform absence of incident response capabilities across this domain is unsurprising given that cyberbiosecurity is a new concept for these laboratories, and incident response planning requires the organizational foundations such as designated personnel, policies, and training, that were themselves identified as gaps. Notably, incident response planning emerged as a top priority in the action plans laboratories developed during the subsequent in-person dialogue.

### 3.3 Universal and critical gaps

Two universal gaps (no laboratory could affirm; 7/7 and 12 critical gaps (6/7 laboratories could not affirm) were identified across Tab 6 (Table 3).

**TABLE 3:** Universal and critical cyberbiosecurity gaps.

Question ID	Question text	Section	Severity
6.1.1.4	Have you identified key hazards at the physical-digital interface for your facility?	Risk evaluation	Universal (7/7)
6.2.2.2	Does your facility have a cyberbiosecurity incident response plan?	Planning & mgmt	Universal (7/7)
6.2.1.2	Does someone in your facility have cyberbiosecurity as part of their roles and responsibilities?	Planning & mgmt	Critical (6/7)
6.2.1.11	Does facility staff undergo regular cyber, data, and information security awareness training?	Planning & mgmt	Critical (6/7)
6.2.2.1	Does your facility have a cyberbiosecurity threat mitigation SOP or policy?	Planning & mgmt	Critical (6/7)
6.2.3.2	Are staff trained on the risks stemming from IoT devices, generative AI chatbots, and other online platforms?	Planning & mgmt	Critical (6/7)
6.2.3.3	Are staff trained on the risks in connecting personal devices to the enterprise system or network?	Planning & mgmt	Critical (6/7)
6.3.2.5	Is staff web browser and application access to the internet restricted?	Infrastructure	Critical (6/7)
6.3.4.2	Do you utilize recognized security standards for the storage, protection, and transfer of sensitive data?	Infrastructure	Critical (6/7)
6.3.5.1	Do you have an end-to-end data flow map for laboratory and pathogen data?	Infrastructure	Critical (6/7)
6.3.5.2	Is the LIMS system and results accessible via an additional health information system?	Infrastructure	Critical (6/7)
6.4.0.2	Is the law enforcement agencies' alert process clearly outlined in the incident response plan?	Incident response	Critical (6/7)
6.4.0.3	Does your facility utilize evaluation policies (after-action reviews) for cyberbiosecurity incidents?	Incident response	Critical (6/7)
6.4.0.4	Are there mechanisms in place for disseminating lessons learned from cyberbiosecurity incident evaluations?	Incident response	Critical (6/7)

The distribution of these gaps is notable: of the 14 universal and critical gaps, six fall in Planning and Management (governance and training), four in Infrastructure and Systems (technical controls and data stewardship), three in Incident Response, and one in Risk Evaluation. Figure 4 shows that 12 of the 14 (86%) are classified as Operational questions rather than Technical questions, and this concentration identifies with severity; both universal gaps are Operational. This pattern reveals that the primary challenge for laboratories entering the cyberbiosecurity domain is operational rather than technical: the personnel designations, policies, training programs, and institutional structures required to manage cyberbiosecurity risks must be established before technical controls can be effectively implemented or sustained.

**FIGURE 4:**
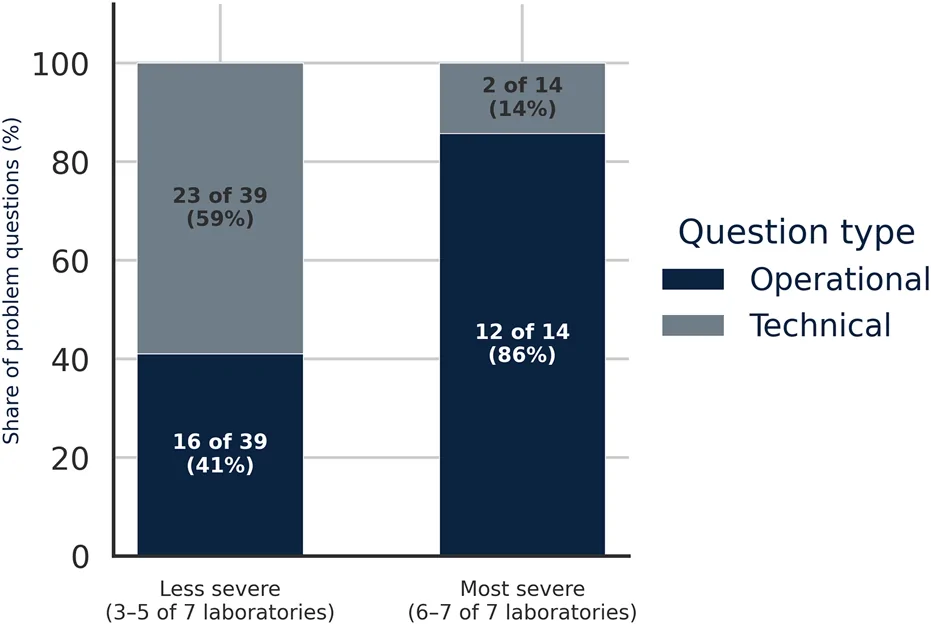
Operational share of cyberbiosecurity problem questions by severity band: among the most sever gaps (universal and critical), 12 of 14 (86%) address Operational capacities; among less severe problem questions 16, of 39 (41%) are Operational.

### 3.4 “No” and “N/A” response patterns

Figures 5, 6 present the distribution of cyberbiosecurity questions across subsections and the share of question laboratories could not affirm, aggregated across laboratories so that gaps cannot be linked to individual facilities. Gap concentrations were highest in Roles and Responsibilities (6.2.1; 10 missed questions), Partnerships and Research Collaborations (6.1.2), IT Systems (6.3.3), Access Controls (6.3.2) and Network System Administration (6.3.4). These patterns indicate that gaps span both operational and technical domains, reinforcing the finding that cyberbiosecurity deficits are multidimensional.

**FIGURE 5:**
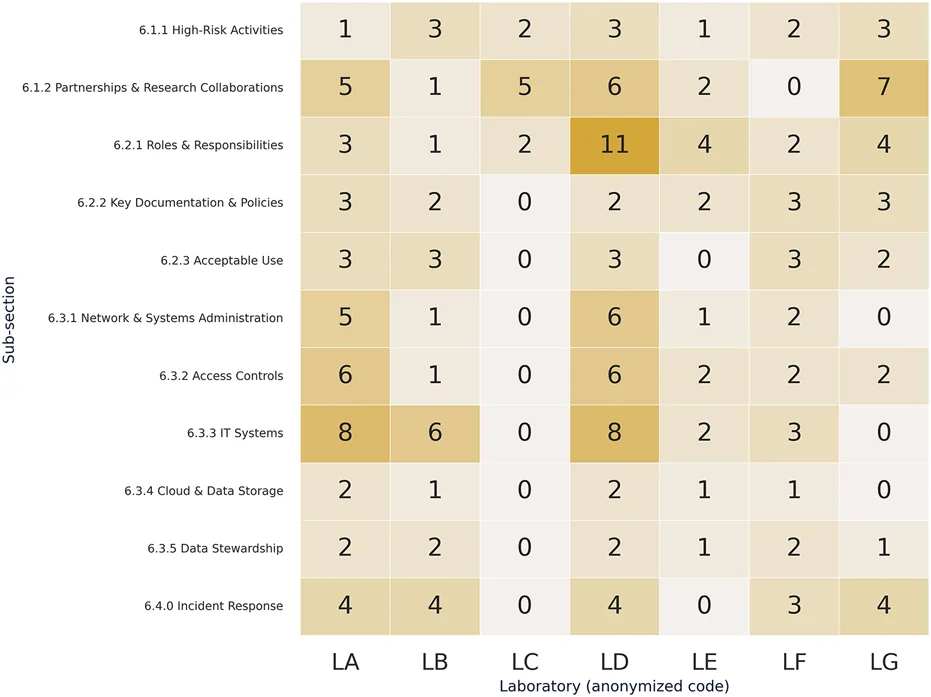
Heatmap of “No” response counts by cyberbiosecurity sub-section and laboratory: darker gold indicates higher counts. The deepest concentation appear in Roles and Responsibilities (6.2.1), IT Systems (6.3.3), and Partnerships and Research Collaborations (6.1.2), and one laboratory (LD) accounts for the largest single cell with 11 counts.

**FIGURE 6:**
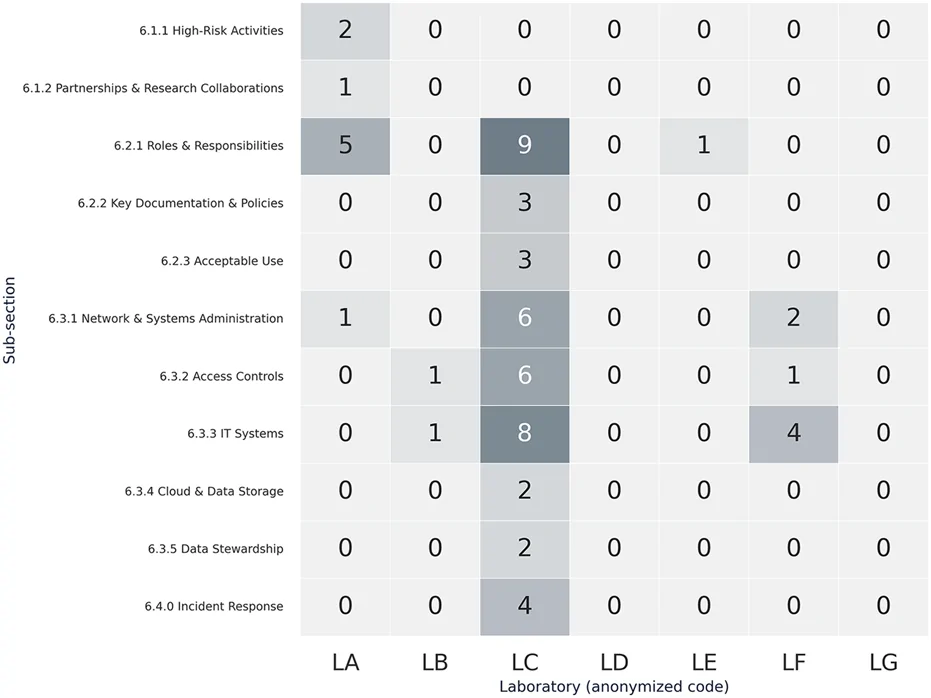
Heatmap of “N/A” responses by cyberbiosecurity sub-section and laboratory: Darker grey indicates higher counts. “N/A′ responses concentrate in IT systems (6.3.3) Network and Systems Administration (6.3.1) and Roles and Responsibilities (6.2.1), driven largely by one laboratory (LC), consistent with a cybersecurity governance, IT administration, and data security being managed at the institutional level, beyond laboratory visibility, for some participants.

The “N/A” responses were concentrated in Roles and Responsibilities (6.2.1) and, for laboratories with institutionally managed information technology, in Network and Systems Administration (6.3.1). This pattern likely reflects institutional complexity: cybersecurity governance, IT administration, and data security may be managed at the university or facility level rather than within the laboratory itself. While this institutional arrangement does not necessarily indicate a lack of capacity, it does suggest fragmented responsibility and limited laboratory-level visibility into cybersecurity practices, itself a potential vulnerability, as laboratory personnel may not be aware of the security measures (or lack thereof) applied to their data and systems.

### 3.5 Corrective actions and priorities

Four of the seven laboratories identified corrective actions in the cyberbiosecurity domain, within the tool, and all seven contributed to the collective master action plan developed during the in-person dialogue. Among these, one laboratory demonstrated the most comprehensive approach, assigning priority levels, estimated implementation timelines, and responsible parties for each proposed action. Across all laboratories that proposed corrective actions, the highest concentration of actions addressed questions in the high-risk activities (Section 6.1.1) and key documents and policies (Section 6.2.2) subsections.

All questions classified as critical or universal were associated with a proposed corrective action from at least one laboratory, with a single exception: Question 6.3.5.2 (LIMS accessibility via additional health information systems), for which no laboratory proposed a corrective measure. Representative corrective actions included:

- Risk Evaluation: Implement a basic cybersecurity analysis, including identifying critical interfaces and main dangers between physical-digital systems (Q6.1.1.4, universal priority)
- Planning and Management: Integrate cyberbiosecurity into the biosafety officer’s or IT service function responsibilities (Q6.2.1.2); train staff regularly and organize at least one annual training session on cybersecurity awareness and data privacy (Q6.2.1.11); finalize and implement an SOP and policy regarding cyberbiosafety and establish a procedure for mitigating cybersecurity threats (Q6.2.2.1); finalize and schedule an incident response plan (Q6.2.2.2, universal priority)
- Infrastructure and Systems: Restrict use of browsers and monitor network activity (Q6.3.2.5); apply security standards such as ISO 27001 and MD5 checksums (Q6.3.4.2); establish a comprehensive data management framework (Q6.3.5.1)
- Incident Response: Define law enforcement notification processes (Q6.4.0.2); organize regular post-incident analyses (Q6.4.0.3); create structured communication mechanisms for lessons learned (Q6.4.0.4)

Three laboratories did not identify any cyberbiosecurity corrective actions, and some that did propose actions failed to address specific questions where they had recorded critical “No” responses. During the November dialogue, participants collaboratively developed a master action plan (Table 4) that assigned specific timelines, priority levels, responsible parties, and linkages to ISO/IEC 27001 sections for each identified gap. Six of the eight action items were designated as HIGH priority, with implementation timelines ranging from one to 6 months. Responsible parties were distributed across laboratory leadership, designated cyberbiosecurity officers (to be appointed), and institutional information systems departments (DSI). Notably, the action plan linked each corrective action to a relevant ISO/IEC 27001 section, including Leadership, Support, Operation, Planning, Context of Organization, and Achievements, providing laboratories with a clear pathway from gap identification to standards-based remediation.

**TABLE 4:** Dialogue master action plan.

Gap identified	Action(s)	Responsible party	Priority	ISO 27001 linkage	Timeline
Lack of cyberbiosecurity awareness and responsibility	Appoint a responsible official for each lab; train person on topic(s)	Lab leadership/directorate	HIGH	Leadership	3 months
Use of personal emails for work	Communication memo/notice of policy against use of personal emails; quarterly reminders	Cybersecurity officer; directorate	MEDIUM	Support	1 month; annual refresher
Noncontrolled/unsecured data sharing	Data sharing protocols; data encryption systems; stakeholder approval to buy hard drive	Cybersecurity officer; lab directors	HIGH	Support	1 month; annual refresher
Lack of visibility of data transfer to subcontractors	Anonymize data; encrypt data; develop confidentiality contract with subcontractors	Lab directors/leadership	HIGH	Context of organization & planning	3–6 months
Lack of training for lab personnel on cyberbiosecurity	Training program for cyberbiosecurity	Directorate of labs; cyberbiosecurity officer	HIGH	Support & operation	Immediate; annual planning
Cyber not incorporated into annual lab plans	Integrate cyberbiosecurity into lab policies	Lab directors; cyberbiosecurity officer	HIGH	Achievements	3 months
Staff not trained on cyberbiosecurity (admin, IT, others)	Training and awareness activities	DSI; cyberbiosecurity officer	HIGH	Planning	3 months
Absence of IT manager	Device management system	DSI	MEDIUM	Planning	3–6 months

### 3.6 Dialogue outcomes

#### 3.6.1 Collective SWOT analysis

During the in-person dialogue, laboratories collectively developed a SWOT analysis focused on cyberbiosecurity and information security management (Table 5). Key strengths identified included the existing legal framework (Law 05–20 and ISO/IEC 27001), quality management system processes already in place at accredited laboratories, and the newly established linkages with DGSSI. Weaknesses centered on lack of budget, lack of qualified personnel, absence of formalized cyberbiosecurity policies, and resistance to change among internal users. Opportunities highlighted international cooperation and training programs, the ongoing digitalization of the Moroccan laboratory system, and the willingness of the Moroccan government to support cyberbiosecurity across agencies. Threats included the rapid evolution of cyber threats, the potential use of AI for attacks, and the risk that diversification across laboratory sectors could impede the development of unified cyberbiosecurity standards.

**TABLE 5:** Collective SWOT analysis from in-person dialogue.

Strengths	Weaknesses	Opportunities	Threats
Law 05–20/ISO 27001 framework	Lack of budget	International cooperation programs and training	Regulatory developments outpacing implementation
National cybersecurity standard exists	Lack of qualified personnel	Digitalization of moroccan laboratory system	Evolution and sophistication of cyber threats
QMS processes in place at accredited labs	Lack of awareness on data management	Use of AI to track and detect threats	AI used for attacks
Newly established linkages to DGSSI	No formalized cyberbiosecurity policy	Improve credibility of moroccan labs internationally	Diversification of sectors makes unified plans difficult
Lab network now versed in BRM	Resistance to change by internal users	Willingness of moroccan government to support	Disruption to medical systems/activity
BRM can now integrate cyberbiosecurity	No surveillance of internet use in work environments	Use of NGOs (e.g., BMAM) to advocate	Insufficient time from authorities to plan/implement

#### 3.6.2 Strategic objectives

Participants developed a collective strategic plan organized around a shared vision: “National implementation of cyberbiosecurity for Moroccan laboratories,” with a mission statement emphasizing resilience and the ability to implement continuity during crisis. Notably, participants opted to develop a collective strategic plan applicable across all participating facilities rather than working in laboratory-specific groups, reflecting a shared recognition that cyberbiosecurity challenges in Morocco require coordinated, sector-wide responses rather than isolated institutional efforts. Three strategic objectives were identified:

1. Comply with national cybersecurity laws and standards, including ISO/IEC 27001
2. Establish cyberbiosecurity policies supported by top management at each institution, with interoperable policies across laboratories
3. Position Morocco as a regional cyberbiosecurity reference center for the Maghreb and broader region

## 4 Discussion

### 4.1 Principal findings

This study presents, to our knowledge, the first use of the Cyber-SLAT as a self-administered laboratory assessment in North Africa. The pilot implementation of the Cyber-SLAT across seven Moroccan laboratories demonstrated that traditional biorisk management functions (Tabs 1-5) are generally operational, underscoring the importance of and results of sustained investments in training, resources, and guidance related to biorisk management; cyberbiosecurity (Tab 6) represents a distinct, emerging domain. The most severe gaps concentrate in operational rather than technical capacities: of the 14 universal and critical gaps identified, 12 (86%) address gaps associated with a gap in personnel, policies, training, and incidence response planning. Second, the assessment process itself generated value beyond its diagnostic function: it was accompanied by a cross-functional dialogue between laboratory and IT personnel who had not previously collaborated on security issues and establishing, for the first time, direct linkages between the laboratory biorisk management community and Morocco’s national cybersecurity authority.

### 4.2 Operational foundations before technical controls

Of the 14 universal and critical gaps identified in this assessment, 12 (86%) are operational: no designated cyberbiosecurity personnel (6/7 laboratories), no threat mitigation policies (6/7), no staff training on cyber and data security (6/7), and no incident response plans (7/7). This concentration of gaps in operational rather than technical efforts carry a practical implication that cyberbiosecurity literature has largely overlooked. Organizational foundations such as assigning responsibility, developing policies, training staff, and establishing incident response procedures are not merely preconditions for more sophisticated technical controls. They are, in themselves, effective countermeasures against the cyberbiosecurity threats that laboratories are most likely to encounter. This finding aligns with evidence from the broader information security literature that effective cybersecurity depends on organizational culture, assigned responsibility, and policy structures before technical investment (Von Solms and Van Niekerk, 2013), and that cybersecurity failures in healthcare settings are predominantly attributable to organizational and human factors rather than technical infrastructure deficits (Kruse et al., 2017). This finding is consistent with efforts of an independent Cyber-SLAT assessment in the DRC which likewise found laboratories constrained by governance and institutional culture rather technological capacities (Kabamba-Tshikongo et al., 2026).

The dialogue risk assessment exercise illustrates this point. When participants applied a structured likelihood-impact methodology to their own laboratory contexts, risks of greatest concern included data breaches via subcontractors; unauthorized data access through shared printers and copiers; and equipment theft. By contrast, phishing received the lowest risk score. The threats that laboratories identified as most critical are addressable through the same organizational measures that the assessment flagged as absent: data-sharing protocols with subcontractors, acceptable use policies for shared devices, designated personnel to oversee data security, and staff training on basic cyber hygiene. A laboratory that designates a cyberbiosecurity officer, trains its staff, restricts personal device use on sensitive networks, and develops an incident response plan has addressed the bulk of its practical threat surface, even if it has not implemented the technically sophisticated controls that dominate the cyberbiosecurity literature. The path to cyberbiosecurity begins with operational foundations and institutional best practices, not technology, and best practices paired with training covers more ground than the field has recognized.

### 4.3 The Cyber-SLAT as methodology

The Cyber-SLAT is, to our knowledge, the first assessment tool that integrates cyberbiosecurity into an existing biorisk management framework rather than treating cybersecurity and biosecurity as separate domains. This design choice has both conceptual and practical significance. Conceptually, the integration of cyberbiosecurity as Tab 6 within a comprehensive biorisk management assessment operationalizes the argument pushed by growing cyberbiosecurity literature that biosecurity and cybersecurity “must work collaboratively and will not be effective working separately” (Murch et al., 2018). By situating cyberbiosecurity questions alongside traditional biorisk management domains, the tool communicates that digital security is a component of sound laboratory management. The comparative analysis enabled by this integrated structure provides empirical evidence for a claim that has previously been made only theoretically: that cyberbiosecurity lags substantially behind traditional biorisk management even in laboratories where conventional biosafety and biosecurity functions are well established. Practically, the integrated approach leverages existing institutional relationships and workflows. Laboratories are already familiar with self-assessment processes for biorisk management; the Cyber-SLAT extends this familiar methodology to a new domain rather than requiring separate assessment infrastructure. The bilingual design (English/French) and spreadsheet-based format were deliberately chosen to minimize technical barriers to adoption in resource-constrained settings where international biosafety guidance documents often lack implementation specificity. The Cyber-SLAT fills this methodological gap by providing a practical, accessible tool that laboratories can complete with existing personnel, using the self-assessment modality already familiar from biosafety practice.

### 4.4 Capacity building in resource-constrained settings and policy implications

The capacity-building methodology employed in this project, combining structured assessment with targeted training, government engagement, and action planning, offers a replicable model for cyberbiosecurity interventions in resource-constrained settings. Several design elements proved particularly effective:

Local partnerships as force multipliers: the partnership with BMAM was not merely a convenience for recruitment and logistics; it was essential for trust-building, cultural competence, and sustainability. BMAM’s credibility within the Moroccan laboratory community facilitated candid self-reporting on sensitive vulnerabilities. Their established relationships with laboratory directors enabled rapid engagement despite project delays. BMAM’s ongoing presence in the Moroccan laboratory ecosystem provides a mechanism for sustained cyberbiosecurity awareness and peer learning that does not depend on continued external funding. This partnership model may be replicable in other LMIC contexts where professional biosafety or biorisk management associations maintain trusted relationships with laboratory networks.

Low-cost interventions first: participating laboratories consistently identified budget limitations and competing priorities as barriers to cyberbiosecurity investment. The action planning framework developed during the dialogue deliberately addressed these constraints by helping laboratories identify interventions that could be implemented with existing resources, such as designating cyberbiosecurity responsibilities within existing staff roles, developing basic policies, institutional best practices, and SOPs, conducting risk and vulnerability assessments, and establishing training programs that integrate cyberbiosecurity into existing biorisk management curricula. These organizational interventions require minimal financial investment but establish the governance foundations upon which more resource-intensive technical controls can later be built.

The findings from this pilot assessment carry implications for multiple stakeholder groups. For laboratories, the most immediate priority is establishing basic organizational foundations: designating personnel with cyberbiosecurity responsibilities, developing incident response plans, and initiating staff training on cyber, data, and information security. These governance measures require minimal financial investment and can be integrated into existing biorisk management structures. For the WHO and international guidance, the findings demonstrated both the relevance of the 2024 Laboratory Biosecurity Guidance’s call for cybersecurity integration into biorisk management and the scale of the implementation challenge (World Health Organization, 2024). No participating laboratory had begun to address the guidance’s recommendations for cybersecurity oversight, cybersecurity risk assessment, or information security protocols, not because they disagreed with these recommendations, but because they lacked awareness, tools, and models for implementation. For donors and implementing partners, the project demonstrates that cyberbiosecurity capacity building in LMIC settings is both feasible and needed. The relatively modest investment, tool development, two engagements, and partnership with a local professional association, produced measurable outputs including completed assessments, identified gaps, action plans, government engagement, and institutional developments. For the cyberbiosecurity field, this study provides the further empirical data on how the theoretical concerns articulated in the literature manifest in actual laboratory practice (Cova et al., 2026). The finding that the most severe gaps are overwhelmingly operational challenges leading cyberbiosecurity literature which has disproportionately emphasized frontier technical threats (Fouad, 2024). The LMIC data complement the field’s predominantly Western evidence base and demonstrate that cyberbiosecurity is not solely a concern for high-resource research institutions, but a global challenge requiring globally inclusive solutions.

### 4.5 Limitations

This study has some notable limitations. First, the sample of seven laboratories, while heterogeneous in institutional type, represents a small and purposively selected group within a single country. The findings may not be generalizable to other LMIC contexts with different governance structures, laboratory capacities, or cybersecurity environments. Second, all findings are based on self-reported assessments without independent verification; although training on the tool was provided before use and in-person support was available from BMAM throughout the assessment period to reduce differences in interpretation, over-reporting, under-reporting, and inconsistent interpretation between institutions remain possible. Third, the Cyber-SLAT’s cyberbisoecurity domain is a preliminary instrument: although it was developed by subject-matter experts, formal content validity, reliability, and inter-rater agreement were not evaluated. Fourth the interpretation of “N/A” responses as non-affirmation is purposive choice designed to assist in capacity-building efforts but may overstate gaps across laboratories. Fifth the cross-sectional design captures a single point-in-time snapshot and cannot assess changes in cyberbiosecurity capacity over time or in response to the capacity-building intervention. Finally, the corrective action analysis relies on stated intentions rather than observed implementation; whether laboratories follow through on proposed actions remains to be assessed.

### 4.6 Future directions

Several research and programmatic directions emerge from this work. Longitudinal follow-up with participating laboratories would assess whether the capacity-building intervention produces sustained improvements and whether action plans are implemented as designed. Expanded deployment of the Cyber-SLAT to additional countries would generate a larger evidence base for cross-national comparison and enable identification of context-specific versus universal cyberbiosecurity challenges. Further, the design team aims to build a hazard metric for Cyber-SLAT questions to allow for more nuisance in the “Yes/No/NA” framing of the tool.

## 5 Conclusion

This study presents, to our knowledge, the first pilot of the full Cyber-SLAT in North Africa, and formally introduces the Cyber-SLAT as a practical tool for integrating cyberbiosecurity into existing biorisk management frameworks. The pilot across seven Moroccan laboratories reveals that cyberbiosecurity is a new domain for operational laboratories, one that the field’s predominantly theoretical literature or existing guidance has not equipped them to address. The finding that severe gaps are operational (designated personnel, policies, training, incident response) carries a clear implication: the path to cyberbiosecurity begins with operational foundations, not technology. For technical controls to be sustainable, they must be built on institutional foundations.

The Moroccan laboratories that participated in this study did not simply reveal gaps; they responded to them. All seven completed the assessment, assembled multidisciplinary teams, and contributed to a collective master action plan linked to ISO/IEC 27001 and articulated a collective strategic vision for cyberbiosecurity in their country and region. These are not the actions of laboratories that are failing; they are the actions of a professional community engaging constructively with a new domain when given the practical tools to do so. The Cyber-SLAT, the capacity-building methodology, and the partnership with BMAM offer a replicable model for initiating cyberbiosecurity capacity building in other LMIC contexts where professional biorisk management networks can serve as trusted intermediaries.

As biological research increasingly depends on digital infrastructure, integrating cyberbiosecurity into biorisk management is essential for protecting sensitive biological data and maintaining the integrity of research and diagnostic activities. The laboratories, researchers, and public health systems of low- and middle-income countries face some of the same cyber threats as their counterparts in high-resource settings. This study provides an initial evidence base and a practical methodology for ensuring that cyberbiosecurity capacity building is globally inclusive and grounded in empirical assessment rather than theoretical speculation.

## Data Availability

The datasets presented in this study can be found in online repositories. The names of the repository/repositories and accession number(s) can be found below: The Cyber-SLAT assessment tool is available from the Johns Hopkins Data Repository, https://doi.org/10.7281/T1DWDA4M. Individual laboratory assessment data are not publicly available. Participating laboratories consented to gap identification by the study team for capacity-building purposes.
