# Supplementary material for: Evaluating laboratory cyberbiosecurity capacity in Morocco: development and pilot implementation of the cyber self-laboratory assessment tool (Cyber-SLAT)
Source: Front Bioeng Biotechnol. 2026 Oct 21;14:1947925. doi: 10.3389/fbioe.2026.1947925 (PMC13731334; doi:10.3389/fbioe.2026.1947925)
Supplement: Supplementary file 1 [file Table1.docx]

*Supplementary Material for: Evaluating Laboratory Cyberbiosecurity Capacity in Morocco: Development and Pilot Implementation of the Cyber Self-Laboratory Assessment Tool (Cyber-SLAT)*

**Supplementary Table S1**: A landscape review of frameworks, guidance, and instruments was conducted to position the Cyber-SLAT against existing approaches discussed in the main text: cybersecurity reference frameworks (NIST CSF 2.0; ISO/IEC 27001:2022), international guidance integrating cybersecurity into biorisk management (WHO, 2024), and laboratory assessment instruments (BioRAM; BSS LAT). No existing option combines the properties required for the target setting: free and downloadable, bilingual (English/French), laboratory-specific, containing dedicated cyberbiosecurity content, integrated into a validated biorisk management instrument, usable offline as a self-assessment, and linked to standards-based action planning.

| **Type** | **Resource** | **Lab-specific** | **Cyberbiosecurity content** | **Cost & access** | **Self-assessment** | **Low-resource fit** |
| --- | --- | --- | --- | --- | --- | --- |
| Framework | NIST CSF 2.0 (2024) | No | Generic cybersecurity | Free | Self-guided | Requires security staff |
| Framework | ISO/IEC 27001:2022 | No | Generic ISMS | Costly certification | Audit-based | High expertise bar |
| Guidance | WHO Laboratory Biosecurity Guidance (2024) | Yes | Yes (conceptual) | Free | No instrument | Guidance only |
| Instrument | BioRAM (Sandia) | Yes (biorisk) | No | Restricted access | Yes (software) | Software dependent |
| Instrument | WHO BSS LAT | Yes (biorisk) | No | Free spreadsheet | Yes, auto-scored | Designed for LMIC laboratories |
| Instrument | Cyber-SLAT (this work) | Yes | Yes, dedicated 57-question domain integrated into a biorisk tool | Free, downloadable, offline | Yes, plus action-plan generation | Designed for low-resource laboratories |
